# Thermodynamic Behavior of Erythromycin Thiocyanate Dihydrate in Six Pure Solvents and Two Binary Solvents

**DOI:** 10.3390/molecules30112424

**Published:** 2025-05-31

**Authors:** Jin Feng, Xunhui Li, Lianjie Zhai, Peizhou Li, Ting Qin, Na Wang, Lu Zhou, Baoxin Zhang, Ting Wang, Xin Huang, Hongxun Hao

**Affiliations:** 1 National Engineering Research Center for Industrial Crystallization Technology, School of Chemical Engineering and Technology, Tianjin University, Tianjin 300072, China; 2022207473@tju.edu.cn (J.F.); 2022207441@tju.edu.cn (X.L.); tqin@tju.edu.cn (T.Q.); x_huang@tju.edu.cn (X.H.); hongxunhao@tju.edu.cn (H.H.); 2 Xi’an Modern Chemistry Research Institute, Xi’an 710065, China; trihever0210@126.com; 3 College of Chemistry and Chemical Engineering, Hainan University, Haikou 570228, China; 15942368170@163.com; 4 Yili Chuanning Biotechnology Co., Ltd., Yili 835000, China; zhoulu@klcnsw.com (L.Z.); zhangbaoxin@klcnsw.com (B.Z.)

**Keywords:** erythromycin thiocyanate dihydrate, thermodynamic behavior, solubility

## Abstract

Thermodynamic parameters play a crucial role in analyzing and optimizing crystallization processes. In this investigation, the solubility profiles of erythromycin thiocyanate dihydrate were determined gravimetrically under atmospheric pressure (0.1 MPa) across six monosolvent systems (methanol, n-propanol, methyl acetate, ethyl acetate, propyl acetate, and water) and two binary solvent mixtures (water–methanol and water–n-propanol), spanning a temperature range of 278.15–318.15 K. The results showed that the solubility of erythromycin thiocyanate dihydrate is apparently affected by temperature and solvent type. For pure solvents, erythromycin thiocyanate dihydrate has higher solubility in alcohol solvents, and lower solubility in ester solvents and water. In mixed solvent systems, erythromycin thiocyanate dihydrate exhibits reduced solubility with higher water content. The experimental solubility values in monosolvent systems were correlated using the Apelblat, Yaws, and Van’t Hoff models, with the Apelblat model showing the best fitting effect. The Apelblat model, Apelblat Jouyban Acre model, and CNIBS/R-K model were employed for data correlation in binary solvent systems, with the Apelblat model and CNIBS/R-K model showing better fitting results.

## 1. Introduction

The solubility data of solutes are among the most important thermodynamic properties, providing crucial information for the development and optimization of processes in fields such as chemical engineering, materials, and pharmaceuticals [1,2,3]. Taking the crystallization process as an example, solubility data and other thermodynamic data, such as dissolution thermodynamic properties, are of great significance for the design and improvement of crystallization processes [4,5,6].

**Figure 1:**
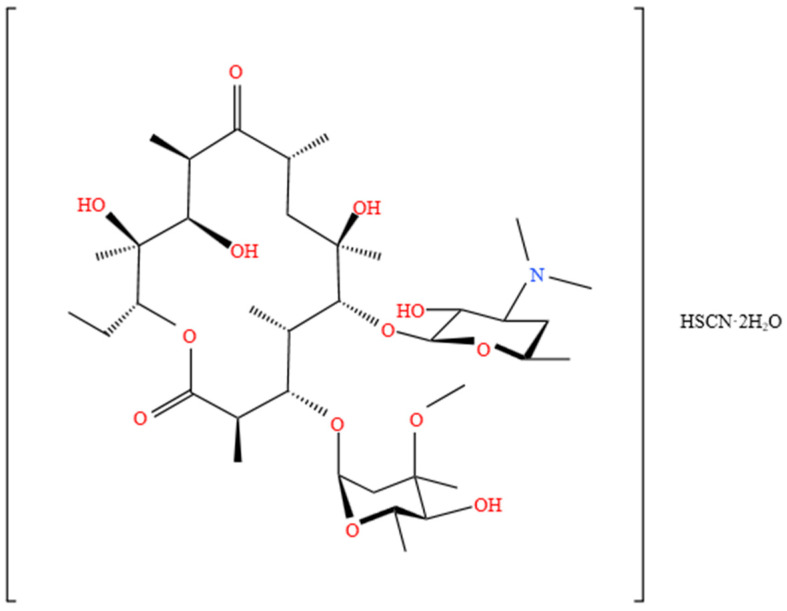
Structural formula of erythromycin thiocyanate dihydrate.

Erythromycin thiocyanate dihydrate is mainly used to treat infections caused by Gram-positive bacteria and mycoplasma in animals. More importantly, it is used as an intermediate for the production of macrolide antibiotics such as erythromycin and azithromycin [7,8,9]. Its structural formula is shown in Figure 1. The selection of solvent plays a critical role in crystallization processes, as it directly impacts both the economic efficiency of the operation and the quality of the final crystalline product. The selection of solvents for industrial applications is generally related to economic value, safety, environmental impact, etc. The alcohols and esters used as solvents in this study have good biodegradability, reducing the risk of long-term environmental residue and posing lower toxicity to humans. They are unlikely to form persistent organic pollutants (POPs) in the environment, and their final degradation products have minimal environmental impact. The current solvent selection has balanced experimental feasibility with EHS (environmental, health, and safety) requirements. Alcohols, esters, and water are commonly employed as solvents or antisolvents in the crystallization of erythromycin thiocyanate dihydrate. However, comprehensive thermodynamic data for this compound in the solvent system remain notably scarce. The lack of thermodynamic data on erythromycin thiocyanate dihydrate in the solvent system commonly used in industrial production makes it difficult to optimize the crystallization process of the drug, which is not conducive to the industrial production of the drug. So far, only Huang [10] has studied the thermodynamic data of erythromycin thiocyanate dihydrate in some solvents. The data gap on the solubility of erythromycin thiocyanate dihydrate is still large. Therefore, this study focuses on the measurement and analysis of the solubility data of erythromycin thiocyanate dihydrate in the solvent system related to alcohol solvent, ester solvent, and water.

Solubility data for erythromycin thiocyanate dihydrate were determined gravimetrically (0.1 MPa) in six monosolvents (methanol, n-propanol, methyl acetate, ethyl acetate, propyl acetate, and water) and two binary mixtures (water–methanol and water–n-propanol) across temperatures ranging from 278.15 to 318.15 K. For monosolvent systems, solubility values were correlated with the Apelblat, Yaws, and Van’t Hoff models. In binary solvent mixtures, the Apelblat, Apelblat–Jouyban–Acree, and CNIBS/R-K models were utilized for data modeling. The thermodynamic data of erythromycin thiocyanate dihydrate in the solvent system related to alcohol solvent, ester solvent, and water were measured and analyzed in this study, which provides a reference for the optimization of the production process of erythromycin thiocyanate dihydrate and the analysis of the solubility data of macrolide antibiotics.

## 2. Results and Discussion

### 2.1. Powder X-Ray Diffraction(PXRD) Data

To prevent phase alterations during the dissolving process, the undissolved solute was taken out for powder X-ray diffraction (PXRD) analysis in each experiment. The PXRD patterns displayed in Figures S1 and S2 confirm that the PXRD characteristic peaks for all experimental samples remain consistent, indicating that the solute did not undergo a phase transition throughout the dissolution process (see in Supplementary Materials). This stability is crucial for validating the solubility characteristics and ensuring reliable outcomes in subsequent analyses.

### 2.2. DSC and TGA Data

The thermal decomposition pathway of erythromycin thiocyanate dihydrate was profiled through differential scanning calorimetry (DSC) and thermogravimetric analysis (TGA), capturing enthalpy transitions and mass loss profiles under controlled heating regimes. The heat flow curve and weight curve are shown in Figure S3. From Figure S3, it can be seen that the initial weight loss of the sample occurs in the range of 332–395 K, and the weight loss ratio is 4.57%, which is close to the theoretical water content of erythromycin thiocyanate dihydrate of 4.34%. After the dehydration phase, the sample began to decompose at 439 K.

### 2.3. Solubility of Erythromycin Thiocyanate Dihydrate in Pure Solvent

**Figure 2:**
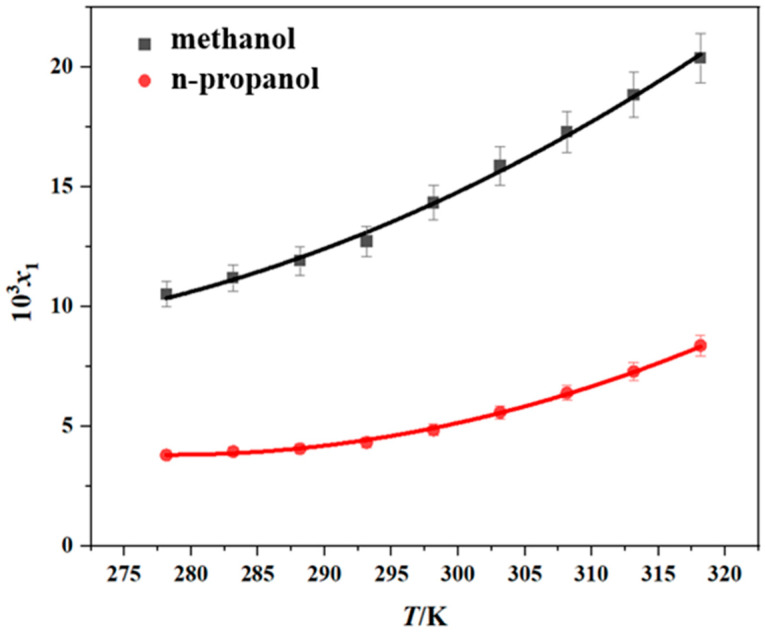
Solubility of erythromycin thiocyanate dihydrate in two pure solvents (data points: experimental values; curves: the Apelblat model fitting values).

**Figure 3:**
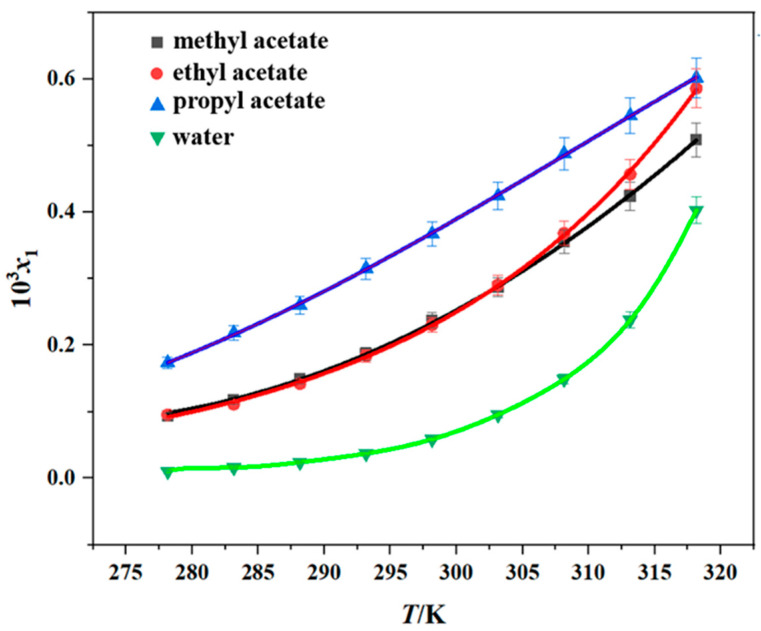
Solubility of erythromycin thiocyanate dihydrate in four pure solvents (data points: experimental values; curves: the Apelblat model fitting values).

The solubility of erythromycin thiocyanate dihydrate was measured in six pure solvents (methanol, n-propanol, methyl acetate, ethyl acetate, propyl acetate, and water) by the gravimetric method over the temperature range of 278.15–318.15 K. The reliability of the experimental approach used in the current research was verified through a comparison between the experimental data from this study and existing literature data [10], and the results are shown in Figures S8 and S9, and Table S1. It is evident from the comparison that the experimental data obtained in this study exhibit minimal discrepancies with the literature values. This low margin of error underscores the credibility and robustness of the measurement techniques utilized herein, thereby affirming the methodological soundness of this research effort. The experimental results obtained are shown in Table 1 and Figure 2 and Figure 3. The experimental data demonstrate that the dihydrate form of erythromycin thiocyanate exhibits enhanced solubility in six distinct pure solvents under elevated temperatures, suggesting that dissolving the compound in these solvents involves heat absorption. Moreover, from Table 2 and Figure 2 and Figure 3, the solubility data demonstrate that methanol exhibits the greatest dissolving capacity for erythromycin thiocyanate dihydrate, with n-propanol showing the second highest value. However, for the other four solvents, such as propyl acetate, ethyl acetate, methyl acetate, and water, the solubility of erythromycin thiocyanate dihydrate is relatively low, with the order of solubility from highest to lowest being propyl acetate > ethyl acetate > methyl acetate > water. The solubility sequence of erythromycin thiocyanate dihydrate in eight solvents (methanol > ethanol > n-propanol > isopropanol > propyl acetate > ethyl acetate > methyl acetate > water) was established using solubility data for ethanol and isopropanol provided in literature [10].

Including the two solvents mentioned in the literature [10], the polarity order of the eight solvents is water > methanol > ethanol > n-propanol > isopropanol > methyl acetate > ethyl acetate > propyl acetate. Within the context of alcohol-based solvents, the solubility of erythromycin thiocyanate dihydrate demonstrates a direct correlation with the increasing polarity of the solvent. Thiocyanate erythromycin dihydrate contains highly polar thiocyanate ions. Alcohols, with both hydrophilic hydroxyl groups and hydrophobic alkyl chains, can encapsulate the hydrophobic macrolide framework of thiacycline erythromycin, reducing intermolecular hydrophobic aggregation and forming a stable solvation layer. The polarity of the ester solvents used in this study is ranked as methyl acetate > ethyl acetate > propyl acetate. The solubility order of erythromycin thiocyanate dihydrate in ester solvents is propyl acetate > ethyl acetate > methyl acetate, indicating that the solubility of solutes in ester solvents decreases with the increase in solvent polarity. This is the opposite of what is found in alcohol-based solvents. When the polarity of esters increases, the van der Waals forces between the alkyl chains of esters and the hydrophobic framework of drugs decrease, leading to a preference for drug molecules to aggregate with each other rather than dissolve. As for water, which processes a high polarity, erythromycin thiocyanate dihydrate has minimal solubility. The extremely high polarity of water significantly increases the solvation energy barrier of hydrophobic frameworks, leading to the precipitation of solutes. Water’s strong hydrogen-bonding ability enhances interactions between drug molecules, favoring the maintenance of a stable dihydrate crystal lattice rather than entering the solvent environment. These experimental outcomes highlight that the solubility of erythromycin thiocyanate dihydrate is significantly enhanced in organic solvents containing hydroxyl groups. The solubility behavior of erythromycin thiocyanate dihydrate presents a noteworthy divergence from the polarity order of different solvents, indicating that the solubility of solutes in solvents cannot be attributed to a single factor. Solute–solvent interactions, hydrogen bond donor/acceptor capacity, and other factors may affect the solubility behavior of solutes in solvents.

The experimental solubility data were modeled with the Apelblat, Yaws, and Van’t Hoff models, as shown in Figure 2 and Figure 3. To assess model accuracy, the ARD and RMSD values were computed and are provided in Tables S2–S4. The average ARD values across all solvents remain below 5%, reflecting satisfactory consistency between calculated solubility values and experimental solubility values of erythromycin thiocyanate dihydrate in the six pure solvents. The Apelblat model exhibited a higher degree of agreement with the experimental results.

### 2.4. Solubility Data of Erythromycin Thiocyanate Dihydrate in Binary Solvents

**Figure 4:**
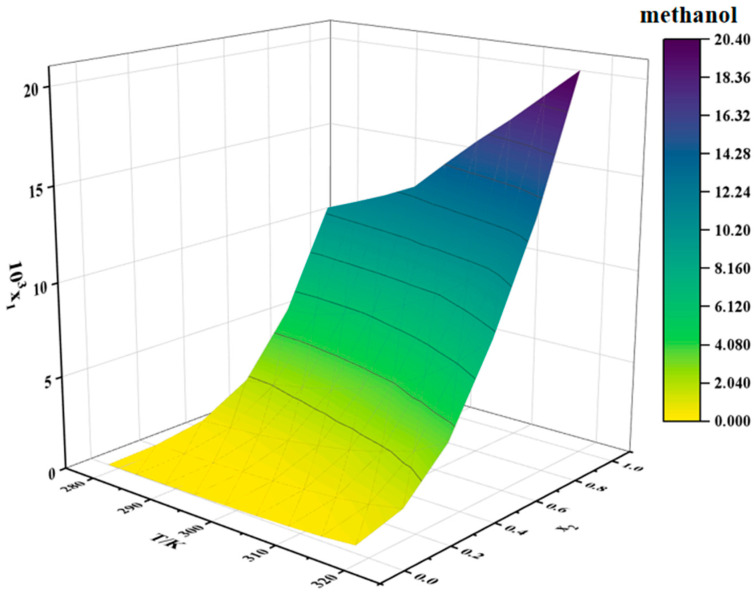
Solubility of erythromycin thiocyanate dihydrate in water–methanol binary solvent.

**Figure 5:**
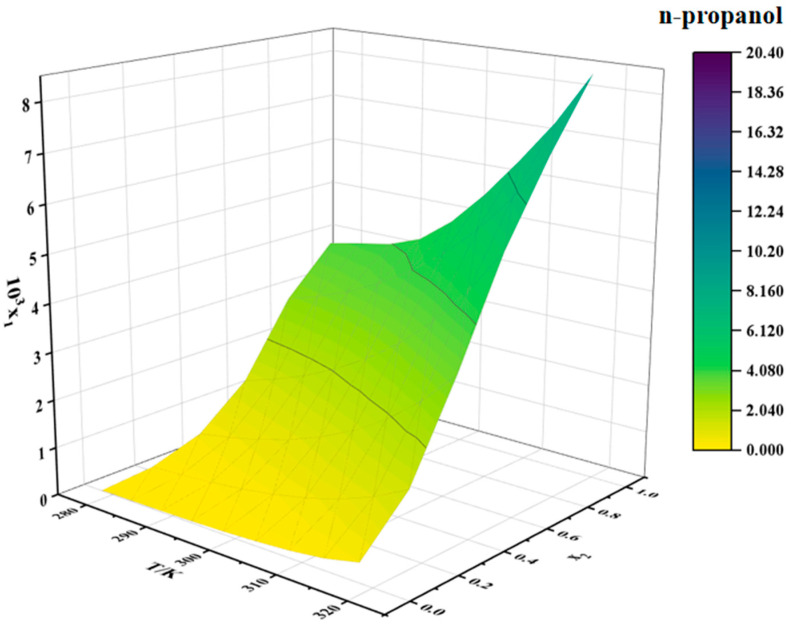
Solubility of erythromycin thiocyanate dihydrate in water–n-propanol binary solvent.

The solubility data for erythromycin thiocyanate dihydrate in water–methanol and water–n-propanol binary mixtures are provided in Table 2 and Table 3, respectively. Two-dimensional solubility curves for erythromycin thiocyanate dihydrate in the binary solvent systems at varying ratios were generated to analyze its solubility behavior, as depicted in Figure 4 and Figure 5.

The solubility of erythromycin thiocyanate dihydrate in water–methanol and water–n-propanol systems shows a direct proportionality to temperature, rising as temperature increases, as shown in Table 2 and Table 3. The solubility data of erythromycin thiocyanate dihydrate in binary solvent systems from this study were integrated and compared with its solubility in water–acetonitrile, water–ethanol, and water–acetone systems reported in the literature [10]. The solubility trends of erythromycin thiocyanate dihydrate in binary systems were observed to vary with water content. In water–acetonitrile and water–acetone mixtures, a distinct pattern of initial solubility enhancement followed by reduction was recorded, with peak solubility achieved at a water mole fraction of 0.5. Conversely, in water–methanol, water–ethanol, and water–n-propanol systems, a gradual decrease in solubility was documented as the initial water content increased across the studied mole fraction range. Noubigh et al. [11] investigated the preferential solvation of pentaerythritol in (methanol, ethanol, and isopropanol) + water mixed solvents, revealing the impact of solvent ratios on pentaerythritol solubility in mixed solvents. The solubility changes of erythromycin thiocyanate dihydrate in mixed solvents can be explained by the concept of preferential solvation. In water–acetonitrile and water–acetone mixed solvent systems, at lower water contents, water shows a stronger tendency for the preferential solvation of erythromycin thiocyanate dihydrate due to its high polarity, which preferentially solvates polar groups of the solute (such as hydroxyl groups), while organic solvents interact with the macrolide structure of erythromycin through hydrophobic effects, forming a synergistic solvation layer, significantly enhancing solubility. Therefore, at lower water contents, the solubility of the solute increases with increasing water content in the solvent. However, once the water content in the solvent reaches a certain level, organic solvents exhibit a stronger tendency for preferential solvation. As the polarity of the mixed solvent gradually approaches that of pure water, the hydrophilic groups of erythromycin thiocyanate become saturated in binding with water, while the hydrophobic regions cannot be effectively stabilized due to insufficient organic solvent proportion, leading to a decrease in solubility. For water–(methanol, ethanol, and n-propanol) mixed solvents, the solubility of erythromycin thiocyanate dihydrate decreases as the water content increases. This is because alcohol solvents act as both hydrogen bond donors and acceptors, forming strong hydrogen bond networks with water, which results in smaller deviations in the preferential solvation around the solute. Increasing the water content directly enhances the overall polarity of the solvent, weakening the stabilizing effect of the alcohol solvents on the hydrophobic structure of erythromycin.

Moreover, under constant temperature, a reduction in the solubility of erythromycin thiocyanate dihydrate is observed with increasing water mole fraction. When the binary solvent composition is close to pure water, the solubility of erythromycin thiocyanate dihydrate in water–methanol binary solvent is generally smaller than that in water–n-propanol and water–ethanol binary solvent. When the binary solvent composition is close to the organic solvent, the solubility of erythromycin thiocyanate in water–methanol binary solvent increases rapidly, which indicates that water has a more obvious negative effect on the dissolution of erythromycin thiocyanate dihydrate in methanol. In high water-content mixed solvents, the hydrogen bond network formed between methanol and water is dense and uniform, resulting in an extremely low proportion of organic solvent molecules preferentially binding around the solute. At this point, the hydrophobic macrolide structure of erythromycin cannot be effectively stabilized by methanol’s weaker hydrophobic interactions, limiting the increase in solubility. The elongation of the carbon chain in alcohols (ethanol-C2 and n-propanol-C3) enhances hydrophobic interactions, providing limited stabilization for hydrophobic groups even at high water content. In contrast, in low water-content mixed solvents, the solvent molecules preferentially binding to the solute surface are mainly methanol, making the preferential solvation effect of methanol on the solute stronger, whereas in ethanol/n-propanol systems, the enhanced hydrophobic interactions may induce the solute molecules to aggregate through hydrophobic stacking, leading to a smaller change in solubility compared to the water–methanol system. It can be inferred that, during the crystallization of erythromycin thiocyanate dihydrate, methanol and water can serve as a suitable solvent and an antisolvent, respectively.

The solubility behavior of erythromycin thiocyanate dihydrate in binary solvent systems was modeled using the Apelblat model, CNIBS/R-K model, and Apelblat–Jouyban–Acree model, with the computational results summarized in Table 3 and Table 4. The parameters and calculation accuracy for these models are provided in Tables S5–S7. The experimental data were validated to align closely with the Apelblat, CNIBS/R-K, and Apelblat–Jouyban–Acree models, with the Apelblat and CNIBS/R-K approaches achieving superior accuracy in fitting the solubility of erythromycin thiocyanate dihydrate.

## 3. Experimental and Computational Methods

### 3.1. Experimental Materials

Erythromycin thiocyanate dihydrate with a purity of 99.0% was provided by Yili Chuanning Biotechnology Co., Ltd. (Yili, China). The solutions were prepared using highly purified deionized water with an electrical resistivity of 18.25 MΩ·cm measured under standard laboratory conditions. The detailed information of the materials used in this work are shown in Table 4.

### 3.2. Characterization Methods

Powder X-ray diffraction (PXRD): The X-ray diffraction pattern of each experimental sample was measured to determine its crystallinity and crystal structure. X-ray diffraction analysis was conducted using a Rigaku D/max-2500 diffractometer (Rigaku, Tokyo, Japan) equipped with Cu Kα radiation (*λ* = 0.71073 Å), with a scan range of 2*θ* = 2–35° at 1 step·s^−1^.

Differential scanning calorimetry (DSC): The melting behavior of erythromycin thiocyanate dihydrate was analyzed via differential scanning calorimetry (Mettler Toledo DSC 1/500, Greifensee, Switzerland) under a nitrogen atmosphere at 70 mL·min^−1^. The sample, weighing approximately 5–10 mg, was heated from 308.15 K to 463.15 K at the rate of 10 K∙min^−1^.

Thermogravimetric analysis (TGA): Thermogravimetric measurements were conducted on the Thermal Analysis System (TGA1, Mettler Toledo, Greifensee, Switzerland). Thermal analysis was carried out on a 5–10 mg sample, with the temperature increasing from 308.15 K to 463.15 K at 10 K∙min^−1^ under a nitrogen atmosphere (flow rate: 90 mL∙min^−1^).

### 3.3. Solubility Measurement of Erythromycin Thiocyanate Dihydrate

The solubility of erythromycin thiocyanate dihydrate was determined gravimetrically in six single-component solvents and two binary solvent mixtures. For each measurement, a certain amount of single solvents and binary solvents were prepared and placed into a small glass EasyMax reactor (Mettler Toledo, Greifensee, Switzerland (*V* = 20 mL)) at the specified temperature. Some erythromycin thiocyanate dihydrate was then added into the reactor until the solid no longer dissolved. The mixture was stirred continuously with magnetic stirring (350 rpm) for 15 h to achieve solid–liquid equilibrium. After establishing phase equilibrium, the solution remained quiescent for three hours and a certain amount of supernatant was filtered into a pre-weighed small beaker (*m*_0_) by preheated syringes and 0.22 μm membrane filters. Subsequently, the small beaker with clear liquid was quickly weighed (*m*_1_) to determine the mass of the clear liquid (*m*_1_ *− m*_0_). Afterwards, the small beaker was placed into an oven with a temperature of 318.15 K to dry the solvent and then was weighed (*m*_2_) to determine the mass of the solute (*m*_2_ *− m*_0_). To enhance measurement precision, three independent determinations were conducted for each solubility point, and the final solubility value was derived from their average. After the solubility determination process of each group of samples was completed, the remaining suspension was filtered to obtain the residual wet solid [12], and the wet solid was tested by the PXRD device.

### 3.4. Solubility Calculation

The mole fraction solubility (*x*_1_) can be obtained through the application of Equation (1):

(1) $$x_{1}=\frac{m_{1}/M_{1}}{\sum_{i=1}^{n}m_{i}/M_{i}}$$

where *m_i_* denotes the mass of each constituent component (solute/solvent), while *M_i_* corresponds to their respective mole masses with units of g∙mol^−1^. Within this formulation, *n* = 2 designates a pure solvent system, whereas *n* = 3 characterizes a binary solvent system in thermodynamic notation.

### 3.5. The Apelblat Model

The Apelblat model is generally considered to be well suited for correlating solubility in various solvents [13]. The methodology is grounded in solid–liquid phase equilibria theory, which takes into account molecular interactions in solution. As a result, it provides a more accurate description of the relationship between solubility and temperature [14].

(2) $$\ln x_{1}=A+B/T+C\ln T$$

In Equation (2), *A*, *B*, and *C* are defined as parameters of the equation, respectively. *T* is the absolute temperature corresponding to solubility.

### 3.6. Yaws Model

The Yaws model, a semi-empirical equation, correlates solubility with temperature [15,16]. This model demonstrates high accuracy in describing solubility data for multiple compounds in various solvents. The Yaws model is shown in Equation (3):

(3) $$\ln x_{1}=A_{1}+\frac{B_{1}}{T}+\frac{C_{1}}{T^{2}}$$

The regression coefficients *A*_1_, *B*_1_, and *C*_1_ in the Yaws thermodynamic formalism are derived through a Levenberg–Marquardt optimization of experimentally acquired (*x*_1_, *T*) dissolution equilibrium pairs, where *x*_1_ represents the solute’s mole fraction in saturated solutions under isobaric conditions and *T* is the absolute temperature corresponding to solubility [17].

### 3.7. Van’t Hoff Model

The Van’t Hoff model is based on the activity coefficient formula theory, which exhibits a thermodynamically governed proportionality linking mole fraction solubility (*x*_1_) to reciprocal temperature (1/*T*). It can predict the solubility at different temperatures and is suitable for a variety of solvent systems [18,19]. It is shown as Equation (4):

(4) $$\ln x_{1}=-\frac{\Delta H_{\mathrm{solu}}}{RT}+\frac{\Delta S_{\mathrm{solu}}}{R}$$

The dissolution equilibrium is parametrized by *x*_1_ (solute mole fraction in saturated phase), *T* (temperature/K), Δ*H*_solu_, and Δ*S*_solu_ (enthalpic and entropic components of dissolution free energy), with *R* as the mole gas constant. Generally, Equation (4) can be transformed to obtain a simplified form of Equation (5).

(5) $$\ln x_{1}=\frac{a}{T}+b$$

The *a* and *b* are Van’t Hoff model parameters.

### 3.8. CNIBS/R-K Model

The CNIBS/R-K thermodynamic framework provides a computational paradigm for predicting dissolution behavior in multi-component solvent systems [20], demonstrating particular efficacy in parameterizing solvation equilibria across binary solvent matrices and ternary co-solvent environments through the explicit incorporation of solvent–solvent interaction potentials, as represented by Equation (6):

(6) $$\ln x_{1}=x_{2}\ln{\left(x_{1}\right)}_{2}+x_{3}\ln{\left(x_{1}\right)}_{3}+x_{2}x_{3}\sum_{i=0}^{N}S_{i}{\left(x_{2}-x_{3}\right)}^{i}$$

In this model, *x*_1_ quantifies the equilibrium solubility as the mole fraction, whereas *x*_2_ and *x*_3_ denote the initial solvent composition ratios in the binary mixed solvent, satisfying the mass balance constraint *x*_2_ + *x*_3_ = 1. *S_i_* represents model parameters, while *N* represents the number of solvent types. In binary mixed solvent systems (*N* = 2), the (1 − *x*_2_) representation can be used in place of *x*_3_, and then Equation (6) can be simplified to Equation (7).

(7) $$\ln x_{1}=B_{0}+B_{1}x_{2}+B_{2}{x_{2}}^{2}+B_{3}{x_{2}}^{3}+B_{4}{x_{2}}^{4}$$

*B*_0_, *B*_1_, *B*_2_, *B*_3_, and *B*_4_ represent model parameters [1].

### 3.9. Apelblat–Jouyban–Acree Model

The Apelblat–Jouyban–Acree model combines the enhanced Apelblat model with the Jouyban–Acree model through systematic integration [21], resulting in a three-dimensional structure that systematically quantifies interactions between temperature, solvent composition, and solute solubility in binary solvent systems. This model excels in scenarios where solvent composition significantly impacts solubility, delivering improved precision in illustrating solubility relationships with solvent composition and temperature [1]. The model expression is shown in Equation (8).

(8) $$\ln x_{1}=x_{2}(A_{1}+\frac{B_{1}}{T}+C_{1}\ln T)+x_{3}(A_{2}+\frac{B_{2}}{T}+C_{2}\ln T)+x_{2}x_{3}\sum_{i=0}^{N}\frac{J_{i}}{T}{\left(x_{2}-x_{3}\right)}^{i}$$

The variable *x*_1_ denotes the solute’s solubility in mole fraction, while *x*_2_ and *x*_3_ indicate the molar concentrations of the two components in the binary solvent mixture. *J_i_*, *A*_1_, *B*_1_, *C*_1_, *A*_2_, *B*_2_, and *C*_2_ represent model constants; *T* (K) represents absolute temperature; and *N* represents the number of solvent types with the values of 0, 1, and 2 for binary solvent systems [22]. The model for the binary solvent systems can be simplified to Equation (9).

(9) $$\ln x_{1}=A_{1}+\frac{A_{2}}{T}+A_{3}\ln T+A_{4}x_{2}+A_{5}\frac{x_{2}}{T}+A_{6}\frac{{x_{2}}^{2}}{T}+A_{7}\frac{{x_{2}}^{3}}{T}+A_{8}\frac{{x_{2}}^{4}}{T}+A_{9}x_{2}\ln T$$

It should be noted that *x*_1_ in Equations (1)–(9) represents the molar solubility of the solute, while *x*_2_ and *x*_3_ in Equations (6)–(9) represent the molar fractions of the two solvents in the binary solvent system. All of these parameters are dimensionless.

### 3.10. Model Evaluation

Several thermodynamic models were employed in this study to correlate experimental data. The goodness-of-fit indicators, specifically the average relative deviation (*ARD*%) and root mean square deviation (*RMSD*), were calculated to assess prediction deviations across the model set [23,24], and they can be expressed as Equations (10) and (11).

(10) $$ARD\%=\frac{100}{N}\sum_{i=1}^{N}\left|\frac{{x_{i}}^{\exp}-{x_{i}}^{cal}}{{x_{i}}^{\exp}}\right|$$

(11) $$RMSD=\sqrt{\frac{1}{N}\sum_{i=1}^{n}{\left(x_{i}^{exp}-x_{i}^{cal}\right)}^{2}}$$

*N* denotes the number of experimental points, while the calculated and experimental solubility data are assigned to *x_i_^cal^* and *x_i_^exp^*, respectively.

## 4. Conclusions

The solubility of erythromycin thiocyanate dihydrate in six pure solvent and two binary solvent systems was measured using gravimetric analysis at temperatures ranging from 278.15 to 318.15 K. A temperature-dependent rise in solubility was observed across all solvents, with alcohol-based solvents exhibiting solubility values that markedly exceeded those of ester solvents and water. In binary systems, an increase in water content at fixed temperatures was found to reduce solubility. The solubility data in pure solvents were analyzed with the Yaws model, Apelblat model, and Van’t Hoff model, where the Apelblat model exhibited the highest correlation accuracy. For binary mixtures, the Apelblat, CNIBS/R-K, and Apelblat–Jouyban–Acree models were applied to the experimental data, with the Apelblat model being identified as the most effective model (Table 3 and Table 4). The solubility data of erythromycin thiocyanate dihydrate obtained in this study further fill the gap in solubility data for erythromycin thiocyanate dihydrate. These data provide a reference for analyzing solubility trends of erythromycin thiocyanate dihydrate and macrolide antibiotics on a broader scale, as well as for improving industrial production processes.

## 5. Associated Content

### Supporting Information

The PXRD patterns and TGA/DSC curves of solids, the parameters and deviations of the Apelblat model, the Yaws model, and the Van’t Hoff model of erythromycin thiocyanate dihydrate in six pure solvents, the parameters and deviations of the Apelblat model, CNIBS/R-K model, and Apelblat−Jouyban−Acree model for erythromycin thiocyanate dihydrate in two binary solvent mixtures, and the experimental values of erythromycin thiocyanate dihydrate in six pure solvents and fitting curves of the Yaws model and the Van’t Hoff model, can be found in the Supporting Information.

## Figures and Tables

**Table 1:** Experimental and calculated solubility values of erythromycin thiocyanate dihydrate in six pure solvents at 278.15–318.15 K (*p* = 0.1 MPa) ^a,b^.

*T*/K	10^3^ *x*_1_^exp^	10^3^ *x*_1_^cal,yaws^	10^3^ *x*_1_^cal,Apelblat^	10^3^ *x*_1_^cal,Van’t^
methanol				
278.15	10.51	9.965	10.31	9.935
283.15	11.19	11.00	11.14	10.98
288.15	11.90	12.10	12.08	12.10
293.15	12.72	13.28	13.13	13.29
298.15	14.33	14.53	14.31	14.54
303.15	15.87	15.85	15.62	15.87
308.15	17.29	17.25	17.10	17.27
313.15	18.83	18.72	18.74	18.75
318.15	20.37	20.27	20.58	20.30
n-propanol				
278.15	3.792	3.264	3.738	3.232
283.15	3.934	3.684	3.894	3.669
288.15	4.058	4.148	4.138	4.147
293.15	4.317	4.657	4.479	4.667
298.15	4.843	5.215	4.931	5.232
303.15	5.582	5.824	5.516	5.843
308.15	6.390	6.489	6.263	6.502
313.15	7.287	7.211	7.210	7.210
318.15	8.369	7.996	8.408	7.970
methyl acetate				
278.15	0.094	0.095	0.092	0.095
283.15	0.118	0.121	0.118	0.121
288.15	0.149	0.151	0.150	0.151
293.15	0.187	0.189	0.189	0.189
298.15	0.237	0.233	0.235	0.233
303.15	0.288	0.287	0.289	0.287
308.15	0.355	0.350	0.352	0.350
313.15	0.424	0.425	0.425	0.425
318.15	0.509	0.512	0.509	0.512
ethyl acetate				
278.15	0.095	0.083	0.092	0.083
283.15	0.111	0.109	0.115	0.109
288.15	0.142	0.142	0.145	0.141
293.15	0.184	0.182	0.182	0.182
298.15	0.231	0.233	0.230	0.233
303.15	0.291	0.296	0.290	0.296
308.15	0.368	0.372	0.366	0.372
313.15	0.457	0.465	0.462	0.465
318.15	0.586	0.577	0.584	0.577
propyl acetate				
278.15	0.174	0.192	0.174	0.192
283.15	0.218	0.226	0.216	0.226
288.15	0.260	0.265	0.263	0.265
293.15	0.315	0.309	0.314	0.309
298.15	0.368	0.358	0.369	0.358
303.15	0.425	0.414	0.426	0.414
308.15	0.488	0.475	0.485	0.475
313.15	0.545	0.543	0.545	0.544
318.15	0.602	0.619	0.603	0.619
water				
278.15	0.010	0.006	0.012	0.006
283.15	0.016	0.011	0.017	0.011
288.15	0.024	0.019	0.025	0.019
293.15	0.037	0.033	0.038	0.032
298.15	0.059	0.055	0.058	0.055
303.15	0.096	0.093	0.092	0.093
308.15	0.149	0.153	0.148	0.153
313.15	0.238	0.248	0.242	0.248
318.15	0.403	0.397	0.402	0.397

^a^ The standard uncertainty of *T* is *u* (*T*) = 0.02 K, and the standard uncertainty of pressure is *u* (*p*) = 0.05 MPa. ^b^ *x*_1_^exp^ is the experimental mole fraction solubility of erythromycin thiocyanate dihydrate, and *x*_1_^cal^ is the calculated mole fraction solubility of erythromycin thiocyanate dihydrate. The relative standard uncertainty of solubility measurement is *u_r_*(*x*_1_^exp^) = 0.05.

**Table 2:** Experimental and calculated solubility values of erythromycin thiocyanate dihydrate in a water–methanol binary solvent mixture at 278.15–318.15 K (*p* = 0.1 MPa) ^a,b^.

x_2_	10^3^ x_1_^exp^	10^3^ x_1_^cal,AJA^	10^3^ x_1_^cal,CNIBS^	10^3^ x_1_^cal,Apelblat^
*T* = 278.15 K				
0	0.010	0.012	0.012	0.012
0.2	0.081	0.074	0.080	0.081
0.4	0.454	0.429	0.454	0.465
0.6	1.852	1.740	1.852	1.861
0.8	5.183	4.825	5.183	5.185
1	10.51	11.87	10.50	10.31
*T* = 283.15 K				
0	0.016	0.017	0.018	0.017
0.2	0.103	0.098	0.102	0.104
0.4	0.550	0.528	0.550	0.557
0.6	2.083	2.000	2.083	2.085
0.8	5.310	5.214	5.310	5.392
1	11.19	12.08	11.19	11.14
*T* = 288.15 K				
0	0.024	0.025	0.027	0.025
0.2	0.137	0.133	0.135	0.137
0.4	0.677	0.662	0.678	0.681
0.6	2.412	2.340	2.412	2.387
0.8	5.777	5.724	5.777	5.751
1	11.90	12.48	11.89	12.08
*T* = 293.15 K				
0	0.037	0.038	0.038	0.038
0.2	0.186	0.183	0.186	0.185
0.4	0.854	0.846	0.855	0.851
0.6	2.791	2.783	2.791	2.789
0.8	6.341	6.378	6.341	6.280
1	12.72	13.05	12.70	13.13
*T* = 298.15 K				
0	0.059	0.058	0.050	0.058
0.2	0.252	0.257	0.260	0.254
0.4	1.121	1.098	1.117	1.085
0.6	3.317	3.359	3.318	3.320
0.8	7.128	7.206	7.128	7.012
1	14.33	13.83	14.30	14.31
*T* = 303.15 K				
0	0.096	0.089	0.094	0.092
0.2	0.357	0.367	0.359	0.356
0.4	1.411	1.447	1.410	1.409
0.6	3.982	4.113	3.982	4.021
0.8	7.971	8.246	7.971	7.990
1	15.87	14.82	15.90	15.62
*T* = 308.15 K				
0	0.149	0.140	0.150	0.148
0.2	0.511	0.531	0.509	0.508
0.4	1.875	1.933	1.876	1.861
0.6	4.973	5.103	4.973	4.950
0.8	9.254	9.550	9.255	9.281
1	17.29	16.05	17.30	17.10
*T* = 313.15 K				
0	0.238	0.222	0.249	0.242
0.2	0.733	0.779	0.714	0.737
0.4	2.438	2.617	2.450	2.496
0.6	6.192	6.407	6.188	6.185
0.8	10.80	11.18	10.80	10.98
1	18.83	17.56	18.80	18.74
*T* = 318.15 K				
0	0.403	0.358	0.381	0.402
0.2	1.088	1.157	1.126	1.087
0.4	3.423	3.585	3.398	3.398
0.6	7.832	8.135	7.843	7.837
0.8	13.30	13.23	13.30	13.20
1	20.37	19.39	20.40	20.58

^a^ The standard uncertainty of *T* is *u* (*T*) = 0.02 K, and the standard uncertainty of pressure is *u* (*p*) = 0.05 MPa. ^b^ *x*_1_^exp^ is the experimental mole fraction solubility of erythromycin thiocyanate dihydrate, *x*_1_^cal^ is the calculated mole fraction solubility of erythromycin thiocyanate dihydrate, and *x_2_* is the initial mole fraction of methanol in binary solvents. The relative standard uncertainty of the solubility measurement is *u_r_* (*x*_1_^exp^) = 0.05.

**Table 3:** Experimental and calculated solubility values of erythromycin thiocyanate dihydrate in a water–n-propanol binary solvent mixture at 278.15–318.15 K (*p* = 0.1 MPa) ^a,b^.

x_2_	10^3^ x_1_^exp^	10^3^ x_1_^cal,AJA^	10^3^ x_1_^cal,CNIBS^	10^3^ x_1_^cal,Apelblat^
*T* = 278.15 K				
0	0.010	0.011	0.009	0.012
0.2	0.100	0.099	0.100	0.100
0.4	0.436	0.424	0.435	0.436
0.6	1.265	1.208	1.265	1.265
0.8	2.806	2.622	2.806	2.806
1	3.792	4.143	3.790	3.738
*T* = 283.15 K				
0	0.016	0.017	0.015	0.017
0.2	0.141	0.137	0.141	0.141
0.4	0.558	0.539	0.558	0.558
0.6	1.480	1.420	1.480	1.480
0.8	3.013	2.865	3.013	3.013
1	3.934	4.231	3.930	3.894
*T* = 288.15 K				
0	0.024	0.026	0.024	0.025
0.2	0.198	0.191	0.198	0.198
0.4	0.716	0.691	0.716	0.716
0.6	1.746	1.688	1.746	1.746
0.8	3.281	3.175	3.281	3.281
1	4.058	4.392	4.060	4.138
*T* = 293.15 K				
0	0.037	0.040	0.039	0.038
0.2	0.276	0.267	0.279	0.277
0.4	0.920	0.893	0.921	0.920
0.6	2.073	2.028	2.073	2.073
0.8	3.619	3.563	3.619	3.619
1	4.317	4.630	4.320	4.479
*T* = 298.15 K				
0	0.059	0.061	0.060	0.058
0.2	0.385	0.375	0.383	0.385
0.4	1.182	1.162	1.183	1.182
0.6	2.478	2.460	2.478	2.478
0.8	4.040	4.047	4.040	4.040
1	4.843	4.949	4.840	4.931
*T* = 303.15 K				
0	0.096	0.094	0.098	0.092
0.2	0.533	0.528	0.531	0.533
0.4	1.521	1.520	1.522	1.521
0.6	2.979	3.010	2.979	2.980
0.8	4.560	4.648	4.560	4.560
1	5.558	5.362	5.560	5.516
*T* = 308.15 K				
0	0.149	0.143	0.153	0.148
0.2	0.734	0.746	0.730	0.735
0.4	1.957	2.001	1.960	1.957
0.6	3.603	3.712	3.601	3.603
0.8	5.201	5.394	5.201	5.201
1	6.390	5.882	6.390	6.263
*T* = 313.15 K				
0	0.238	0.220	0.240	0.242
0.2	1.007	1.057	1.005	1.008
0.4	2.520	2.648	2.522	2.520
0.6	4.378	4.613	4.377	4.378
0.8	5.988	6.319	5.988	5.988
1	7.287	6.526	7.290	7.210
*T* = 318.15 K				
0	0.403	0.338	0.397	0.402
0.2	1.376	1.502	1.385	1.376
0.4	3.245	3.520	3.237	3.245
0.6	5.344	5.772	5.349	5.344
0.8	6.956	7.470	6.955	6.956
1	8.369	7.320	8.370	8.408

^a^ The standard uncertainty of *T* is *u* (*T*) = 0.02 K, and the standard uncertainty of pressure is *u* (*p*) = 0.05 MPa. ^b^ *x*_1_^exp^ is the experimental mole fraction solubility of erythromycin thiocyanate dihydrate, *x*_1_^cal^ is the calculated mole fraction solubility of erythromycin thiocyanate dihydrate, and *x*_2_ is the initial mole fraction of n-propanol in binary solvents. The relative standard uncertainty of the solubility measurement is *u_r_* (*x*_1_^exp^) = 0.05.

**Table 4:** Sources and properties of chemical substances used in this work.

Chemical Name	Mass Purity (%)	Source
erythromycin thiocyanate dihydrate	≥99.0	Yili Chuanning Biopharmaceutical Co., Ltd. (Yili, China)
methanol	≥99.5	Li’an Long Bohua (Tianjin) Pharmaceutical Co., Ltd. (Tianjin, China)
n-propanol	≥99.5	Li’an Long Bohua (Tianjin) Pharmaceutical Co., Ltd. (Tianjin, China)
methyl acetate	≥99.5	Li’an Long Bohua (Tianjin) Pharmaceutical Co., Ltd. (Tianjin, China)
ethyl acetate	≥99.5	Li’an Long Bohua (Tianjin) Pharmaceutical Co., Ltd. (Tianjin, China)
propyl acetate	≥99.5	Li’an Long Bohua (Tianjin) Pharmaceutical Co., Ltd. (Tianjin, China)

## Data Availability

The original contributions presented in this study are included in the article/Supplementary Material. Further inquiries can be directed to the corresponding authors.

## Supplementary Materials

The following supporting information can be downloaded at https://www.mdpi.com/article/10.3390/molecules30112424/s1. Figure S1: PXRD spectra of erythromycin thiocyanate dihydrate raw material and sample undissolved in pure solvents; Figure S2: PXRD spectra of sample undissolved in mixed solvents; Figure S3: TGA/DSC curve of erythromycin thiocyanate dihydrate; Figure S4: Solubility of erythromycin thiocyanate dihydrate in two pure solvents ((scatters: experimental values; curves: the Yaws model fitting values); Figure S5: Solubility of erythromycin thiocyanate dihydrate in two pure solvents (scatters: experimental values; curves: Van’t Hoff model fitting values); Figure S6: Solubility of erythromycin thiocyanate dihydrate in six pure solvents (scatters: experimental values; curves: the Yaws model fitting values); Figure S7: Solubility of erythromycin thiocyanate dihydrate in six pure solvents (scatters: experimental values; curves: the Van’t Hoff model fitting values); Figure S8: Comparison between experimental and literature values of solubility of erythromycin thiocyanate dihydrate in methanol; Figure S9: Comparison between experimental and literature values of solubility of erythromycin thiocyanate dihydrate in n-propanol; Table S1: Comparison of Some Experimental Data and Published Data; Table S2: Parameters of the Apelblat Model of Erythromycin Thiocyanate Dihydrate in 6 Pure Solvents; Table S3: Parameters of the Yaws Model of Erythromycin Thiocyanate Dihydrate in 6 Pure Solvents; Table S4: Parameters of the Van’t Hoff Model of Erythromycin Thiocyanate Dihydrate in 6 Pure Solvents; Table S5: Parameters and Deviations of the Apelblat Model Erythromycin Thiocyanate Dihydrate in Two Binary Solvent Mixtures; Table S6: Parameters and Deviations of the CNIBS/R-K Model for Erythromycin Thiocyanate Dihydrate in Two Binary Solvent Mixtures; Table S7: Parameters and deviations of the Apelblat–Jouyban–Acree Model for Erythromycin Thiocyanate Dihydrate in Two Binary Solvent Mixtures. Reference [10] is cited in the Supplementary Materials.
