# Supplementary material for: Thermodynamic Behavior of Erythromycin Thiocyanate Dihydrate in Six Pure Solvents and Two Binary Solvents
Source: Molecules. 2025 May 31;30(11):2424. doi: 10.3390/molecules30112424 (PMC12156870; doi:10.3390/molecules30112424)
Supplement: Supplementary file 1 [file molecules-30-02424-s001.zip › molecules-3619243-supplementary.pdf]

Supporting Information

Thermodynamic Behavior of Erythromycin Thiocyanate Dihydrate in Six Pure Solvents and Two Binary Solvents

Jin Feng [1], Xunhui Li [1], Lianjie Zhai [2], Peizhou Li [3], Ting Qin [1], Na Wang [1,*], Lu Zhou [4], Baoxin Zhang [4], Ting Wang [1,*], Xin Huang [1] and Hongxun Hao [1]

[1] National Engineering Research Center for Industrial Crystallization Technology, School of Chemical Engineering and Technology, Tianjin University, Tianjin 300072, China; 2022207473@tju.edu.cn (J.F.); 2022207441@tju.edu.cn (X.L.); tqin@tju.edu.cn (T.Q.); x_huang@tju.edu.cn (X.H.); hongxunhao@tju.edu.cn (H.H.)

[2] Xi'an Modern Chemistry Research Institute, Xi'an 710065, China; trihever0210@126.com

[3] College of Chemistry and Chemical Engineering, Hainan University, Haikou 570228, China; 15942368170@163.com

[4] Yili Chuanning Biotechnology Co., Ltd., Yili 835000, China; zhoulu@klcnsw.com (L.Z.); zhangbaoxin@klcnsw.com (B.Z.)

* Correspondence: wangna224@tju.edu.cn (N.W.); wang_ting@tju.edu.cn (T.W.)

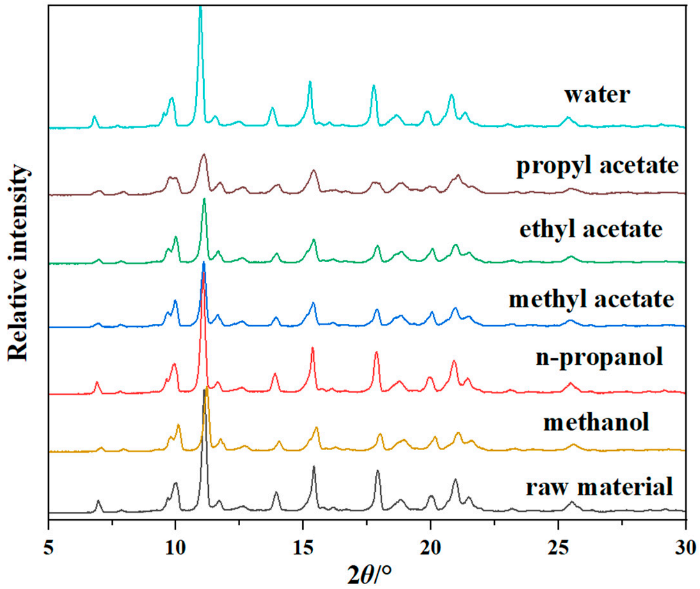

Figure S1. PXRD spectra of erythromycin thiocyanate dihydrate raw material and sample undissolved in pure solvents

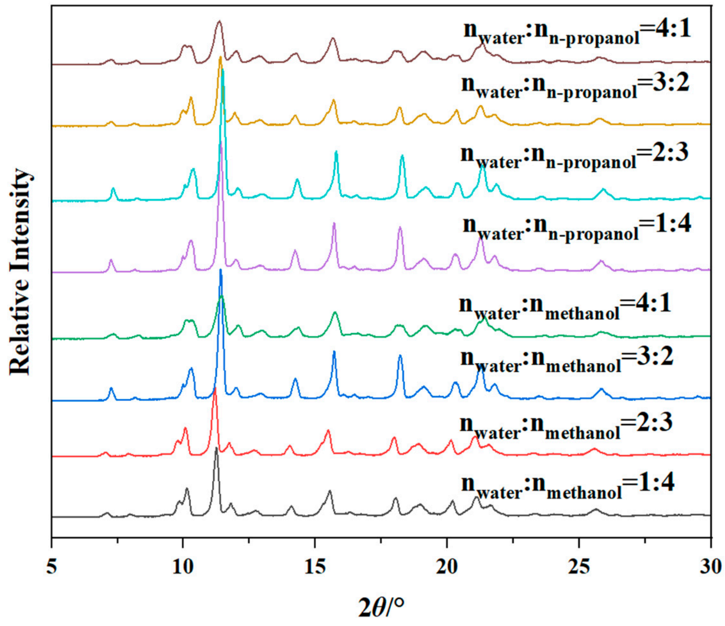

Figure S2. PXRD spectra of sample undissolved in mixed solvents

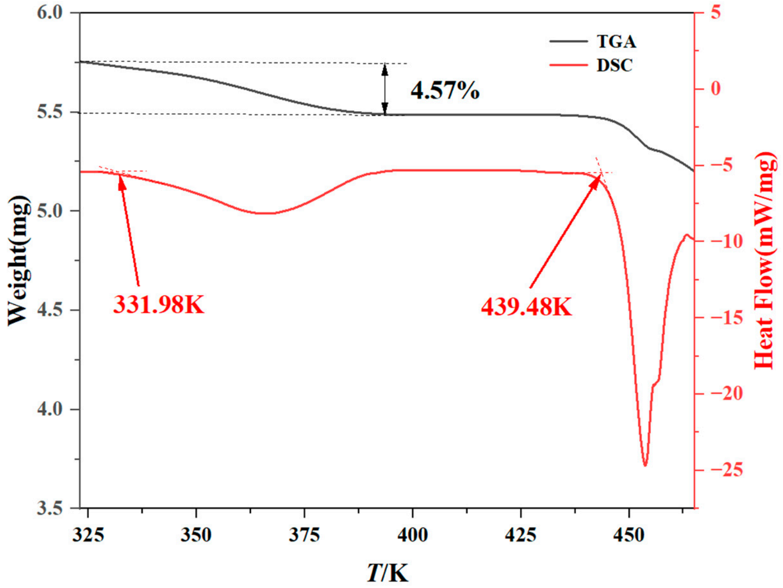

Figure S3. TGA/DSC curve of erythromycin thiocyanate dihydrate

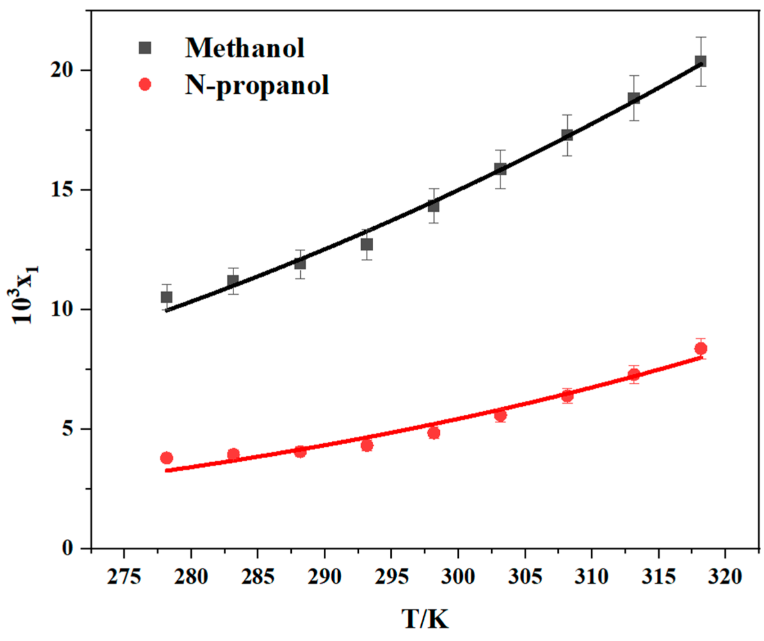

Figure S4. Solubility of erythromycin thiocyanate dihydrate in two pure solvents (scatters: experimental values; curves: the Yaws model fitting values)

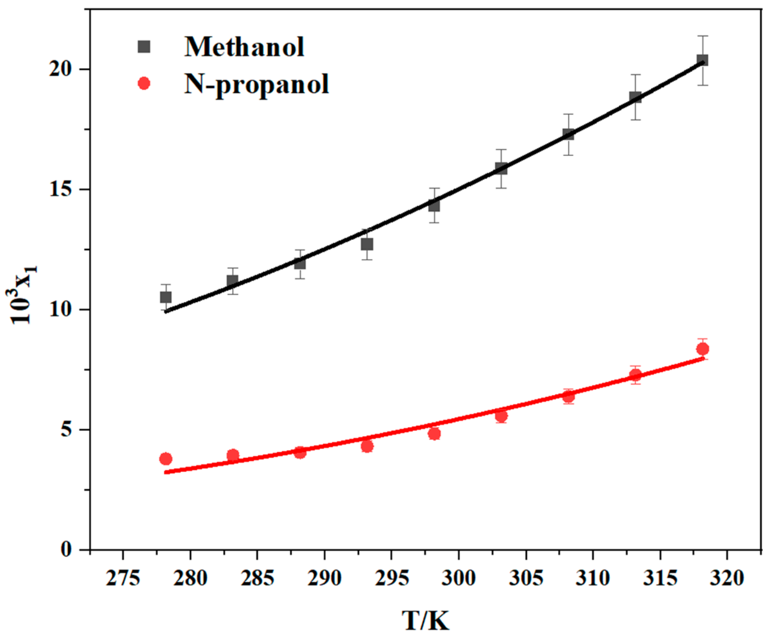

Figure S5. Solubility of erythromycin thiocyanate dihydrate in two pure solvents (scatters: experimental values; curves: Van't Hoff model fitting values)

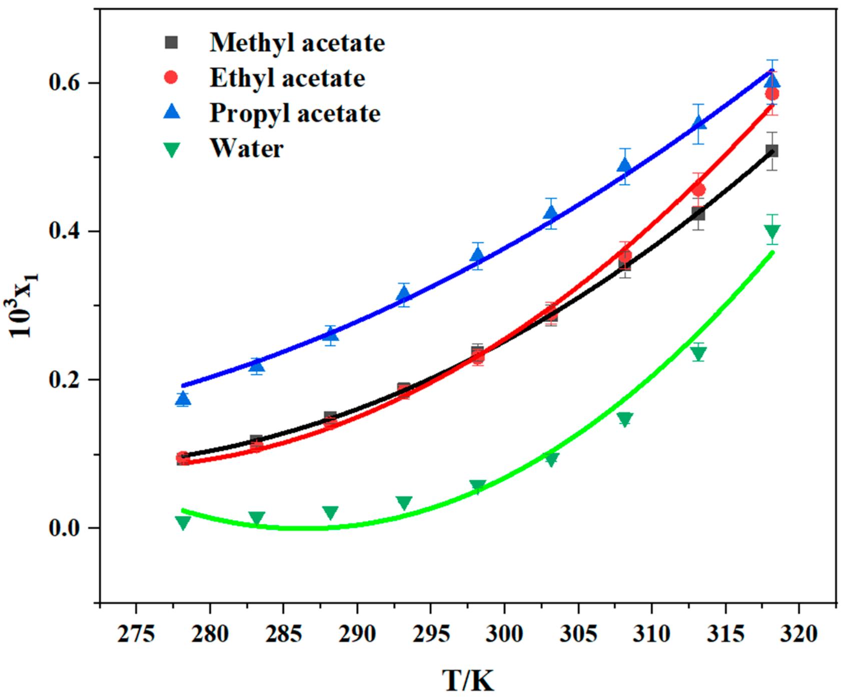

Figure S6. Solubility of erythromycin thiocyanate dihydrate in six pure solvents (scatters: experimental values; curves: the Yaws model fitting values)

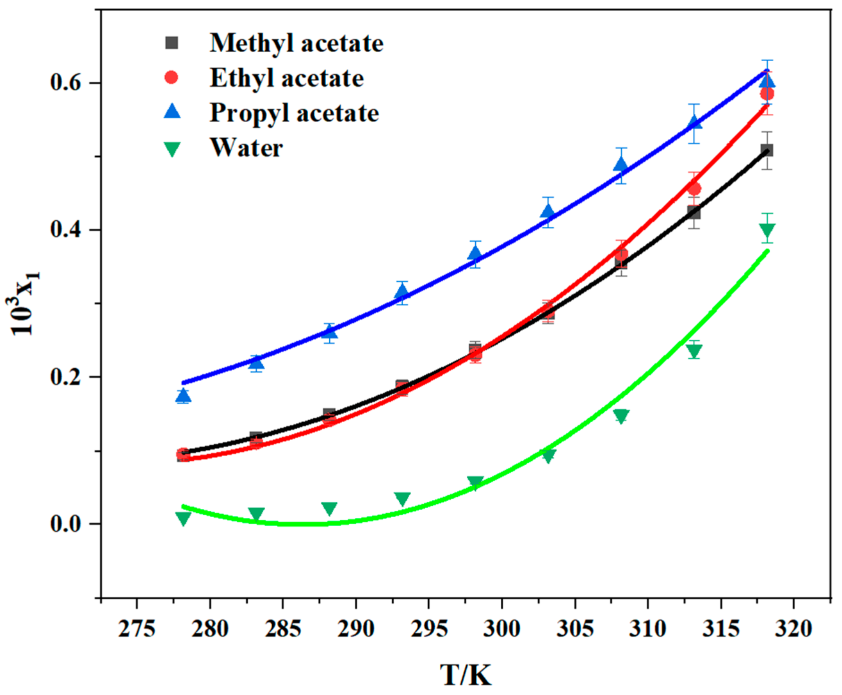

Figure S7. Solubility of erythromycin thiocyanate dihydrate in six pure solvents (scatters: experimental values; curves: the Van't Hoff model fitting values)

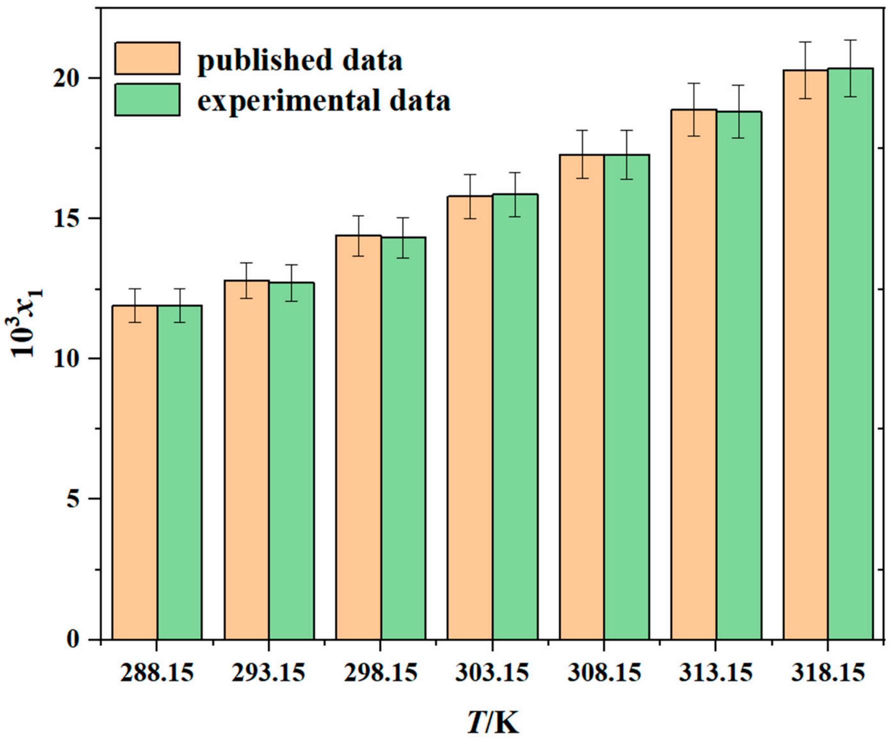

Figure S8. Comparison between experimental and literature values of solubility of erythromycin thiocyanate dihydrate in methanol

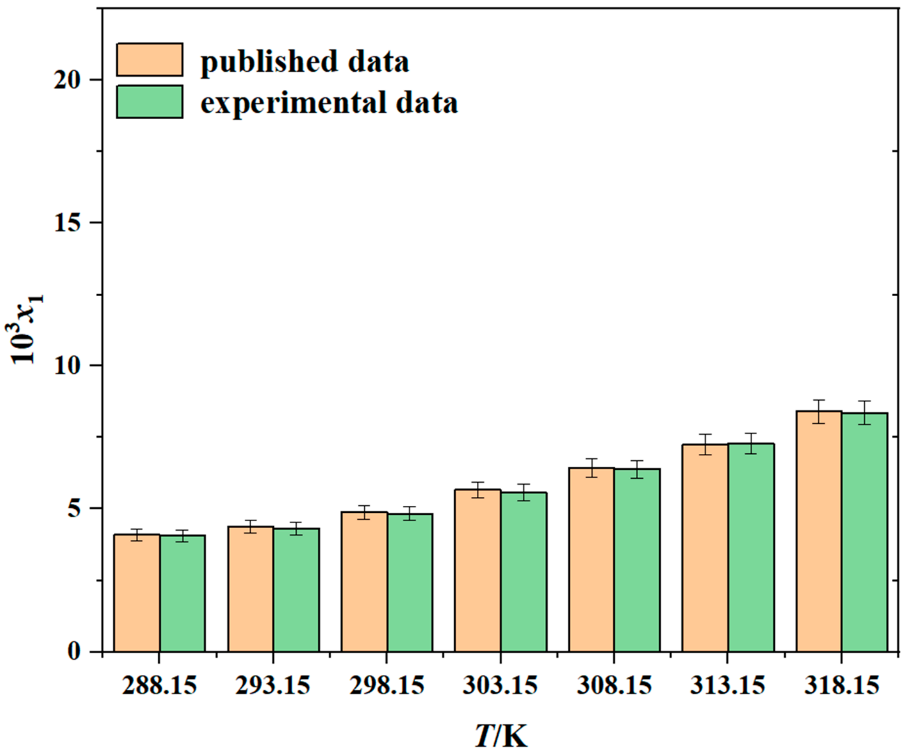

Figure S9. Comparison between experimental and literature values of solubility of erythromycin thiocyanate dihydrate in n-propanol

Table S1. Comparison of Some Experimental Data and Published Data[1]

T/K	$10^3x_1^{exp}$ (published)	$10^3x_1^{exp}$ (this study)	RD%
methanol			
288.15	11.9	11.90	0
293.15	12.8	12.72	0.625
298.15	14.4	14.33	0.486
303.15	15.8	15.87	0.443
308.15	17.3	17.29	0.058
313.15	18.9	18.83	0.370
318.15	20.3	20.37	0.344
n-propanol			
288.15	4.09	4.058	0.782
293.15	4.37	4.317	1.213
298.15	4.88	4.843	0.758
303.15	5.67	5.582	1.552
308.15	6.43	6.390	0.622
313.15	7.25	7.287	0.510
318.15	8.41	8.369	0.488

Table S2. Parameters of the Apelblat Model of Erythromycin Thiocyanate Dihydrate in 6 Pure Solvents

solvent	A	B	C	ARD%	10^4RMSD
methanol	-120.5761	3863.1471	18.1431	1.2701	2.1391
n-propanol	-461.9640	18750.0070	69.1106	1.7065	1.0450
methyl acetate	84.6487	-7354.8089	-11.9939	0.7591	0.0170
ethyl acetate	-207.5026	5359.5577	31.7937	1.4322	0.0290
propyl acetate	266.0351	-14504.7050	-39.5414	0.4917	0.0182
water	-811.0304	28916.9136	123.6125	3.1229	0.0196
average	\	\	\	1.4637	0.5447

Table S3. Parameters of the Yaws Model of Erythromycin Thiocyanate Dihydrate in 6 Pure Solvents

solvent	A_1	B_1	C_1	ARD%	10^4RMSD
methanol	1.4679	-1826.7286	37977.1730	1.8279	2.9509
n-propanol	3.4976	-3231.9196	185435.1169	5.3463	2.9732
methyl acetate	4.1205	-3721.7945	1.7344	1.1398	0.0274
ethyl acetate	6.2836	-4442.5665	22505.8228	2.6431	0.0633
propyl acetate	0.7655	-2593.5675	-82.0665	3.1982	0.1121
water	21.5070	-9334.2183	9.6534	14.5259	0.0562
average	\	\	\	4.7802	1.0305

Table S4. Parameters of the Van't Hoff Model of Erythromycin Thiocyanate Dihydrate in 6 Pure Solvents

solvent	*a*	*b*	*ARD*%	10^4*RMSD*
methanol	13139.3812	8.8965	1.8738	3.075
n-propanol	16599.3220	12.0008	5.6378	3.1379
methyl acetate	30942.8912	34.2576	1.1393	0.0274
ethyl acetate	35702.6174	50.2138	2.6865	0.0641
propyl acetate	21567.7069	6.3731	3.1982	0.1121
water	77689.6991	179.0802	14.6484	0.0562
average	\	\	4.8640	1.0788

Table S5. Parameters and Deviations of the Apelblat Model Erythromycin Thiocyanate Dihydrate in Two Binary Solvent Mixtures

x_1^0	*A*	*B*	*C*	*ARD*%	10^4*RMSD*
		Water-methanol			
0	-811.0304	28916.9136	123.6125	3.1229	0.0196
0.2	-582.6981	23894.1540	87.3345	0.8391	0.7502
0.4	-554.8421	21675.5730	83.6202	0.3778	0.083
0.6	-628.2684	23850.1040	95.0311	1.323	0.1244
0.8	-716.5968	26568.1690	108.6788	0.5197	0.0193
1	-120.5761	3863.1471	18.1431	1.2701	2.1391
		Water-n-propanol			
0	-811.0304	28916.9136	123.6125	3.1229	0.0196
0.2	-137.1500	803.6154	22.2186	0.317	0.0113
0.4	-208.7710	5189.8460	32.4038	0.0022	0.0003
0.6	-282.0160	9542.2970	42.8270	0.0017	0.0007
0.8	-355.1200	13813.7500	53.2289	0.0014	0.0006
1	-461.9640	18750.0070	69.1106	1.7065	1.0450
average	\	\	\	1.0504	0.3511

Table S6. Parameters and Deviations of the CNIBS/R-K Model for Erythromycin Thiocyanate Dihydrate in Two Binary Solvent Mixtures

T/K	B_0	B_1	B_2	B_3	B_4	ARD%	10^4RMSD
				Water-methanol			
278.1500	-11.3454	9.6422	0.7898	-6.2900	2.6470	2.4127	0.0071
283.1500	-10.9519	8.2066	5.4245	-13.5356	6.3645	1.6236	0.0076
288.1500	-10.5283	7.1338	7.7086	-16.4461	7.7008	2.2301	0.0162
293.1500	-10.1827	7.3429	5.5743	-13.7844	6.6838	0.3337	0.0037
298.1500	-9.9064	8.2057	2.1144	-10.2139	5.5526	3.1501	0.0531
303.1500	-9.2671	5.5255	9.0858	-18.6990	9.2134	0.3013	0.0091
308.1500	-8.8051	4.7497	10.5262	-20.5262	9.9983	0.1467	0.0070
313.1500	-8.2965	3.4503	13.2963	-23.5574	11.1334	1.3421	0.1066
318.1500	-7.8735	4.5788	6.7082	-13.7739	6.4683	1.6499	0.2111
				Water-n-propanol			
278.1500	-11.5793	15.3684	-21.5450	21.5636	-9.3831	1.8270	0.0050
283.1500	-11.1054	14.4785	-20.0478	19.4080	-8.2723	1.2367	0.0053
288.1500	-10.6404	13.7070	-19.0579	18.1426	-7.6579	0.0977	0.0006
293.1500	-10.1491	12.7017	-17.3554	16.0213	-6.6630	0.9486	0.0101
298.1500	-9.7147	11.8448	-15.4294	13.0693	-5.1009	0.5400	0.0094
303.1500	-9.2326	10.6261	-12.5295	9.0399	-3.0960	0.4432	0.0132
308.1500	-8.7865	9.6704	-10.3769	5.9948	-1.5548	0.5338	0.0257
313.1500	-8.3342	8.6837	-8.2833	3.2880	-0.2755	0.2127	0.0169
318.1500	-7.8316	7.2664	-5.0338	-0.6098	1.4257	0.4091	0.0559
Average	\	\	\	\	\	1.0799	0.0313

Table S7. Parameters and deviations of the Apelblat–Jouyban–Acree Model for Erythromycin Thiocyanate Dihydrate in Two Binary Solvent Mixtures

A_1	A_2	A_3	A_4	A_5	A_6	A_7	A_8	A_9	ARD%	$RMSD$
					Water-methanol					
-723	25330	110	398	-11598	1826	-4279	2069	-62	4.5343	4.1721
					Water-n-propanol					
-337	8140	53	-37	9340	-4265	3490	-1344	3.11	4.7212	2.4637
Average	\	\	\	\	\	\	\	\	4.6278	3.3179
